# Genomic technologies and the diagnosis of 46, XY differences of sex development

**DOI:** 10.1111/andr.13708

**Published:** 2024-07-31

**Authors:** Firman Idris, Andrew H. Sinclair, Katie L. Ayers

**Affiliations:** ^1^ The Murdoch Children's Research Institute Melbourne Australia; ^2^ The Department of Paediatrics University of Melbourne Melbourne Australia

**Keywords:** 46, XY DSD, diagnosis, genetics, male sex determination, technology

## Abstract

Differences/disorders of sex development can be caused by disruptions to the molecular and cellular mechanisms that control development and sex determination of the reproductive organs with 1:100 live births affected. Multiple genes are associated with 46, XY differences/disorders of sex development that can cause varying clinical phenotypes. An accurate genetic diagnosis is essential to guide clinical care for individuals with 46, XY differences/disorders of sex development and can contribute to family planning. The use of genomics in differences/disorders of sex development has grown, with several advances employed in genetic diagnosis; however, diagnostic rates have stagnated at less than 50% for these conditions. This review will discuss 46, XY differences/disorders of sex development, its molecular causes, and the genomic technologies currently utilized for diagnosis with focus on reports from the last 5 years. We also touch on the challenges in diagnosing 46, XY differences/disorders of sex development and discuss new and future technologies that promise to improved diagnostic rates for these difficult conditions.

## 1 BACKGROUND

The transformation of the embryonic bipotential gonad into a testis or an ovary involves a cascade of events driven by multiple genes and pathways. In mammals, this process is largely determined by sex chromosome complement. Individuals with XX chromosomes will typically develop ovaries, whereas those with XY chromosomes will typically develop testes.^1^ One of the breakthroughs in the field was the discovery of the Y‐chromosome linked *SRY* gene, the molecular switch that triggers male sexual development.^2^ Since this, multiple genes have been associated with sex determination/differentiation, and in many cases, variations in these genes or members of their signaling pathways have been associated with differences/disorders of sex development (DSD).

DSD is an umbrella term to describe congenital conditions where sex development is atypical. Covering a broad variety of phenotypes, the diagnosis and management of DSD is often complicated. However, knowing the genetic cause can help guide clinical management reducing unnecessary and costly examinations that are otherwise part of a long diagnostic odyssey for many of these patients.^3^

Genomic technologies, such as massively parallel sequencing (MPS), have proven to be a valuable diagnostic tool for individuals or families with DSD. They deliver a genetic diagnostic yield of up to 30%–45%,^4^, ^5^ a marked increase from traditional technologies that typically deliver a 15% diagnostic yield.^6^ In this review, we discuss the range of 46, XY DSDs and their molecular causes. We review the use and utility of genomic technologies in the diagnosis of 46, XY DSD, with specific focus on the last 5 years. We examine the importance of noncoding regions and structural genomic variations and discuss the genomic advances that may help to drive diagnostic yields higher in the future.

## 2 DIFFERENCES OF SEX DEVELOPMENT

DSD refers to conditions where the development of chromosomal, gonadal, or anatomical sex is atypical.^7^ DSDs can arise when the complex cellular and molecular processes controlling sex development and sexual differentiation are disrupted. The term covers a wide range of phenotypes that may be identified at birth due to visible abnormalities in the external genital or detected later in life such as at puberty. In recent years, there has also been increase in prenatal diagnoses due to the rise of noninvasive prenatal genetic testing that may identify incongruence between chromosomal/genetic sex and external genitalia on imaging or chromosomal aneuploidies.^8^ The incidence rate of DSD overall can reach as high as 1:100 live births when borderline conditions are included,^3^ although there is significant variability in the incidence of different DSD conditions. DSDs represent a diverse group of genetic conditions affecting 1.7% of births^9^ and accounts for 7.5% of birth defects.^10^ They range from complete 46, XY or 46, XX sex reversal to genital anomalies such as ambiguous genitalia (1 in 4500) or hypospadias diagnosed 1 in 200 boys.^10^, ^11^

DSDs can be classified into three main categories: sex chromosome DSD, 46, XX DSD, and 46, XY DSD.^7^ Sex chromosome DSD includes conditions such as Turner syndrome (45, X), Klinefelter syndrome (47, XXY), or chimerism (i.e., a mix of 46, XX and 46, XY cells). 46, XX DSD encompasses disorders of ovary development and function such as 46, XX ovotesticular (OT) DSD, where a genetic female develops testicular tissue, or congenital adrenal hyperplasia (CAH)—where adrenal cortisol production is impaired. 46, XY DSD includes conditions where hormone production or action is disrupted or where testicular development is absent or aberrant. Diagnosis and management of DSD often require a holistic approach involving endocrinologists, surgeons, geneticists, and psychologists.^12^

## 3 46, XY DSD: a varied phenotype

46, XY DSDs can be further categorized by the underlying biological cause; disruptions to gonadal development; disruptions in androgen synthesis or action (DASA); or other uncategorized variations such as those that affect penis development.^7^ These are further described below.

### 3.1 Disruptions to gonadal development and testicular regression

**FIGURE 1:**
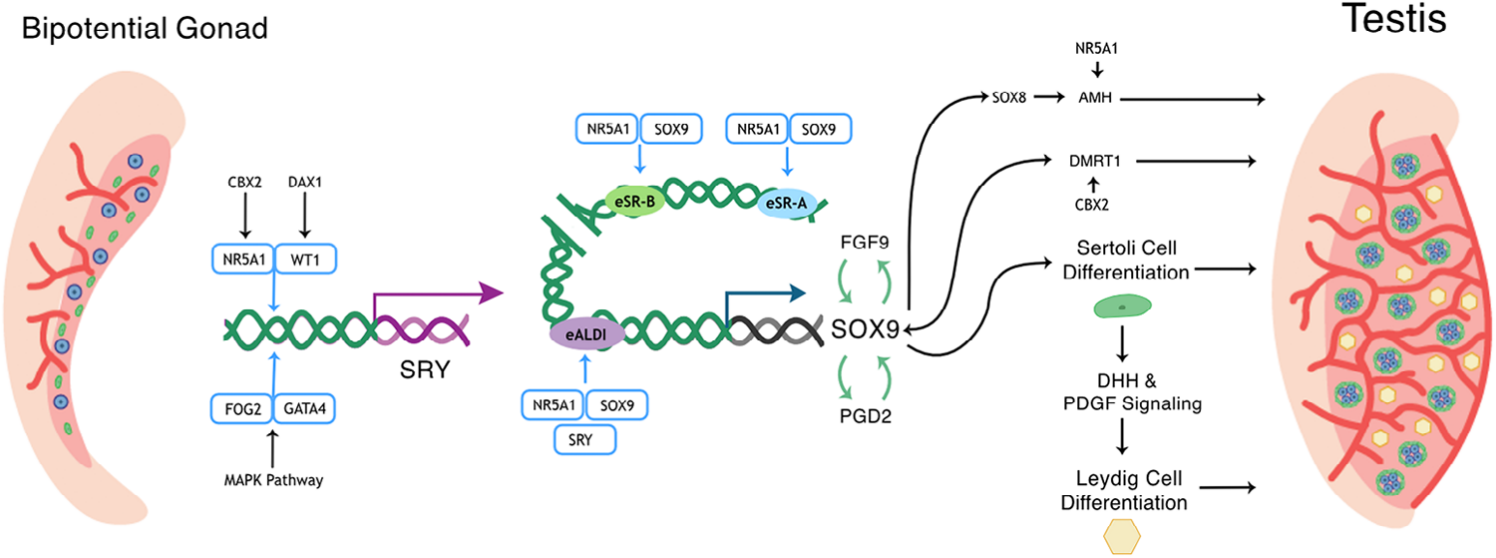
Genes involved in male sex determination in humans. The expression of *SRY* triggers the activation of its downstream target that includes *SOX9*. SOX9 activation induces the development of the testis via Sertoli cells differentiation. These then drive degradation of the Müllerian duct via anti‐Müllerian hormone (AMH) and Leydig cell differentiation through signals such as desert hedgehog (DHH).

The fetal gonad originates from the intermediate mesoderm that forms the urogenital ridge and, subsequently, the bipotential gonad.^13^ In this bipotent tissue, there is initially no observable difference between male and female gonads. Male sexual differentiation initiates through the activation of the transcription factor *SRY* (Figure 1),^14^ which then activates its targets, arguably the most important one of which is *SOX9*,^15^ which induces testicular development and represses ovarian pathway activation. SOX9 expression triggers specification of Sertoli cells.^16^ These cells initiate and maintain testis development, produce anti‐Müllerian hormone (AMH), vital for the degradation of the Müllerian duct,^17^ and secrete factors such as desert hedgehog (DHH), which then trigger differentiation of the androgen‐producing Leydig cells.^18^ These androgens are essential for masculinization of external and internal male structures, including the development of the Wolffian ducts into vas deferens and epididymis.^19^ Therefore, when testis development and function, in particular that of the Sertoli and Leydig cells, is disrupted, both internal and external reproductive tissues are affected.

46, XY gonadal dysgenesis (GD) describes aberrant testis development during embryogenesis. Complete GD (CGD) occurs when there is no gonad development such as in Swyer syndrome. Due to the absence of steroid production, individuals with 46, XY CGD often present externally as typically female and may have a uterus and Müllerian structures. Hormonal evaluation may reveal increased follicle‐stimulating hormone (FSH) and luteinizing hormone (LH) indicative of hypergonadotropic hypogonadism. This condition can be caused by variants in genes that are crucial for testis development such as *SRY*, *SOX9*, *WT1*, and *NR5A1, MAP3K1*, and *DMRT1*.^20^ In some cases, CGD can be accompanied by additional features; for example, in Campomelic dysplasia caused by *SOX9* mutations, individuals also present with skeletal malformation.^21^

Partial GD (PGD) is often associated with a more varied external phenotypes ranging from typical male, ambiguous genitalia to typical female. Depending on the severity of testicular dysgenesis, internal Müllerian structures may be absent or fully developed. Endocrine evaluation can also show hypergonadotropic hypogonadism; however, PGD patients often have elevated gonadotropin levels during infancy and puberty.^22^ Some disorders that are associated with 46, XY PGD are Frasier syndrome, Denys‐Drash syndrome, and alpha‐thalassemia/X‐linked mental retardation syndrome.^23^ Variants that are found to be associated with PGD are reported in genes such as *PBX1*,^24^ *NR5A1*,^25^ *DHH*,^26^ and *HHAT*.^27^ Additionally, structural variants involving duplication of the genetic loci containing, or in proximity to, the *NR0B1* gene have been described in GD cases.^28^, ^29^, ^30^ It is postulated that overexpression of *NR0B1* causes GD through suppression of *SF1*‐mediated transcription.^31^

46, XY OT‐DSD is another condition where early fetal development is disrupted, although in this case both testicular and ovarian tissue may be present. This condition is extremely rare and only accounts for 7% out of all OT‐DSD cases.^32^ Genetic variants in the *NR5A1*,^33^ *DMRT1*,^34^ *SRY*,^35^ and *SOX9* ^36^ have been described in individuals with 46, XY OT‐DSD.

Finally, atrophy or loss of function in testicular tissue after its initial development is referred to as testicular regression syndrome (TRS). This condition can be caused by genetic or endocrine disruptions, torsion during the perinatal and fetal period, or vascular occlusion because of thrombosis. TRS can occur unilaterally or bilaterally, and individuals can present with normal external genitalia but partial or completely absent testicular tissue.^37^ TRS is rare, occurring in around 1:20,000 of baby boys and 0.5%–4.5% of cryptorchid boys.^38^ Individuals with TRS also have varying phenotypes depending on the stage where the testis becomes nonfunctional.^38^ The previous research has described heterozygous variants in *DMRT1, NR5A1, WT1*, and *SOX9* in individuals with TRS.^39^, ^40^ A recent review found that 42% of reported *DHX37* variants occur in individuals with TRS,^41^ though variants in this gene are also linked with other 46, XY DSD phenotypes such as GD.

### 3.2 Disorder of hormone synthesis and action (DASA)

46, XY DSD can be caused by defects in the biosynthesis or action of androgens, specifically in the enzymes involved in conversion of cholesterol to testosterone, or in their receptors.^42^ Examples include recessive variants in the 17‐beta‐hydroxysteroid dehydrogenase 3 gene encoding the enzyme required for the conversion of androstenedione to testosterone. These individuals may present with undescended testes (UDT), the presence of the seminal vesicles, epididymis, vas deferens, and undervirilized or feminized external genitalia.^43^, ^44^ More than 30 variants comprising missense, small deletions, insertions, splice site, and nonsense variants of the *HSDB17B3* have been reported.^45^, ^46^, ^47^, ^48^, ^49^

Another example of DASA is 5‐alpha reductase type 2 (5αRD2) deficiency. This enzyme converts testosterone (T) to its more biologically active form, dihydrotestosterone (DHT), which binds to the androgen receptor (AR) with a higher affinity.^50^ This is an autosomal recessive condition, and more than 100 variants in the *SRD5A2* gene have been reported.^51^ Recent review has noted that 5αRD2 is a condition with no clear genotype–phenotype correlation with affected individuals presenting with varying degree of virilization.^52^ During puberty, patients may develop secondary male sexual characteristics that only require testosterone, such as the deepening of the voice, enlargement of the phallus, and beard growth. As a result, some children that were raised as females may change gender at puberty.^53^

Genetic variants in receptor for T and DHT, AR also cause DSD at a rate of 1 in 99,000 births,^54^ termed androgen insensitivity syndrome (AIS). There are currently more than 600 *AR* genetic variants reported in the human gene mutation database (HGMD).^55^ AIS can be complete (CAIS), partial (PAIS), or mild (MAIS).^56^ In CAIS, the external genitalia appear typically female, and there is no development of the male internal genitalia. Individuals with PAIS present with a more varying phenotype where there can be a degree of undervirilisation, hypospadias, and cryptorchidism (bilateral or unilateral). In these patients, gynecomastia can also occur during puberty. MAIS is typically associated with phenotypically male genitalia and infertility due to lack of androgen effect during spermatogenesis.^57^, ^58^

Unlike androgens, AMH is secreted by the Sertoli cells to control Müllerian duct regression. Recessive variants in *AMH* or its receptor (*AMHR2*) cause a rare form of 46, XY DSD termed persistent Müllerian duct syndrome (PMDS), with only 200 reported cases worldwide before 2021.^59^ PMDS is characterized by the failure of the Müllerian duct to regress during sexual development meaning that duct derivatives, such as the fallopian tubes, cervix, uterus, and the upper vaginal structures, are present in 46, XY males, whereas the development of the male external genitalia is unaffected due to the normal androgen biosynthesis.^60^

### 3.3 Additional DSDs

Hypospadias is a mild DSD characterized by the abnormal placement of the urethral opening. It is classified on the basis of the meatal position, posterior, penile, or anterior.^61^ Hypospadias is one of the most common forms of congenital anomaly, with a prevalence of 1 in 200–300 boys.^11^ It can be caused by a number of factors, including genetics, prenatal hormonal exposure, environmental, and maternal–placental factors. A genetic diagnosis is found in around 30% of the cases with genes such as *WT1, HSD3B2*, and *BMP5* associated.^11^ Like hypospadias, UDT, or cryptorchidism is a common occurrence in 46, XY babies, found in between 15% and 30% of premature and 1%–3% of full‐term male babies.^62^, ^63^ Cryptorchidism is thought to have both a genetic and environmental cause and can very often be successfully treated in surgery called orchidopexy. Just one definitive genetic cause has been associated with familial cryptorchism; recessive variants in the *RXFP2* gene,^64^, ^65^ with biallelic variants with INSL3 also proposed to be causative.^66^ An additional gene that has been associated with hypospadias, micropenis, and/or cryptorchidism is *MAMLD1* (mastermind like domain containing 1).^5^, ^67^, ^68^, ^69^ However, some variants in this gene are also present in unaffected individuals^70^, ^71^ and both in vitro functional studies of missense *MAMLD1* variants^70^, ^72^ and *Mamld1* knockout mouse models^73^ suggest minimal impact on normal male sexual development. Thus, although this gene should not be overlooked in these phenotypes, the exact role and contribution of *MAMLD1* variants to 46, XY DSD require further clarification, and it may act in an oligemic manner with variants in other important genes.^67^, ^68^, ^74^

## 4 THE CURRENT LANDSCAPE OF GENETIC DIAGNOSTICS FOR INDIVIDUALS WITH 46, XY DSD

Technologies for the detection of genetic aberrations have rapidly improved in the last decade, vastly increasing our understanding of the causes of DSD, revealing a vast array of contributing genetic and genomic aberrations. We now know that in addition to chromosomal aneuploidies, causative variants can range from single nucleotide changes to large structural rearrangements and can affect the exonic, intronic, or intergenic genome. The diagnostic journey for individuals with DSD has traditionally followed a stepwise manner with clinical phenotyping and karyotyping followed by endocrine analysis and in some cases genetic testing. However, in recent years, a parallel approach of genetics alongside traditional clinical testing has been increasingly recommended to achieve a faster diagnosis and allow early benefits of this.^75^, ^76^

**FIGURE 2:**
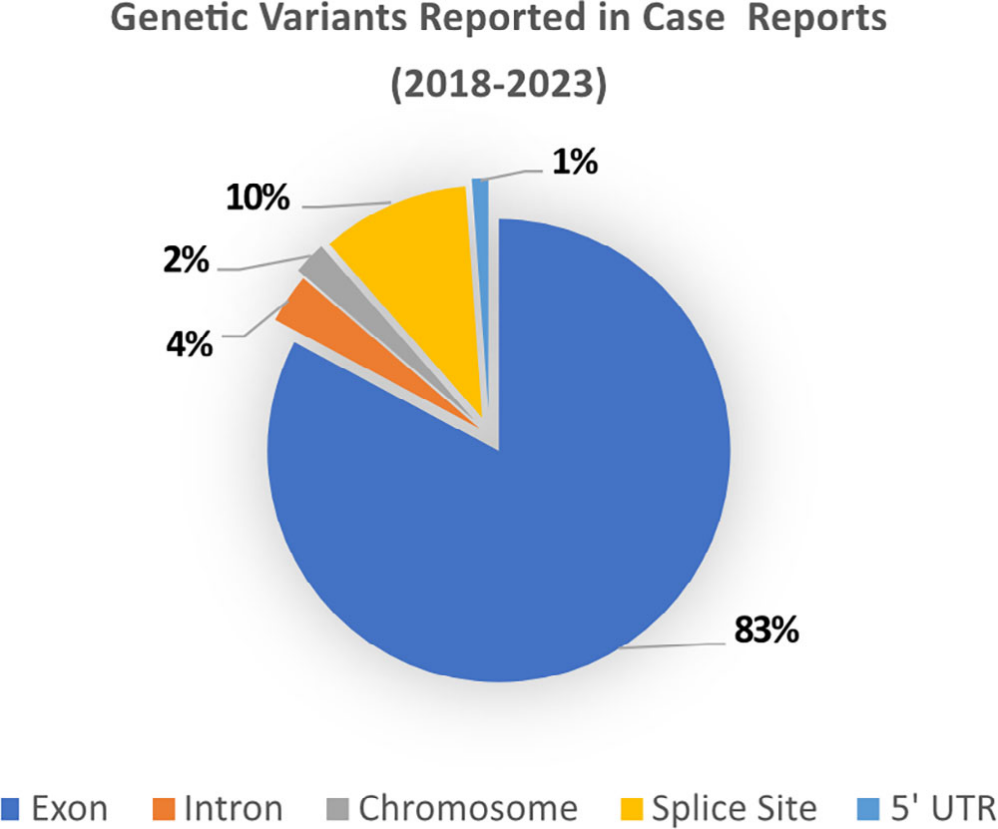
Types of genetic variants found in published case reports from 2018 to 2023 in individuals with 46, XY differences/disorders of sex development (DSD). The majority of variants identified affect an exon/coding region. UTR, untranslated region.

In a review of case reports and cohort studies that employed genetic diagnostics from the last 5 years (2018–2023) (Tables 1 and 2), we found that a wide array of technologies (from Sanger sequencing to long read technologies) had been deployed in the identification of nearly 200 novel variants in known DSD genes and to implicate novel genes such as *BMPR1B, GNAS, GHR*,^77^ *OTX2, PROP1, SOS1*,^78^ and *STARD8*.^79^ Over 80% of variants reported in published case reports affected exonic regions; however, more recent publications have also reported variants in splice sites and noncoding regions such as the 5′UTR and introns (Figure 2). Below, we summarize the technologies applied to DSD diagnostics and how these have evolved.

**TABLE 1:** 46, XY differences/disorders of sex development (DSD) case reports and publications with genetic diagnosis (2018–2023).

Publication	DSD type	Gene reported	Variant found	Location of variant	Zygosity	Year
Martinez et al.^80^	46, XY DSD w/heart defect	GATA4	p.C238R	Exon	Het	2018
	46, XY DSD w/micropenis and UDT		p.W228C			
	46, XY DSD w/micropenis		p.P226L			
Rothacker et al.^81^	46, XY DSD GD	DHH	p.R164P	Exon	Hom	2018
Ilaslan et al.^79^	46, XY DSD GD	STARD8	p.S913N	Exon	Hem	2018
Chen et al.^82^	Leydig cell hypoplasia	LHCGR	p.Q246*	Exon	Hom	2018
			p.R283*			
Sullivan et al.^83^	DASA	HSD17B3	c.277 + 4A > 7	Intron	Hom	2018
Sarathi et al.^84^	DASA	CYP17A1	p.R449C	Exon	Hom	2018
Unal et al.^85^	PMDS	AMRH2	c.233 − 1G > A	Splice site	Hom	2018
			c.233 − 1G > A			
Abd Wahab et al.^86^	OT‐DSD		Mosaic 46, XX/46, XY	Chromosome		2019
Nagy et al.^87^	46, XY DSD GD	NR5A1	Del Exon 5 and 6	CNV	Het	2019
Farnaaz et al.^88^	46, XY DSD GD (syndromic)	PBX1	p.R235Q	Exon	Het	2019
Guran et al.^89^	46, XY DSD GD (syndromic)	PPP2R3C	p.L193S	Exon	Hom	2019
			p.F350S			
			p.L103P			
			p.F350S			
Garcia‐Acero et al.^90^	46, XY DSD GD	NR0B1 dup	Xp dup	CNV	N/A	2019
Fernandez‐Cancio et al.^91^	PMDS	AMRH2	p.G40A	Exon	Hom	2019
Schteingart et al.^92^	PMDS	AMH	c.−225delA	Intron	Hom	2019
Wagner‐Mahler et al.^93^	46, XY DSD micropenis and UDT	GATA4	46, XY, del(8)(p23.1p23.1)	CNV		2019
Al Shamsi et al.^94^	46, XY DSD GD	MAP3K1	p.W657R	Exon	Het	2020
Kunitomo et al.^95^	46, XY DSD GD (syndromic)	LHX9	p.Q316R	Exon	Het	2020
Laan et al.^78^	46, XY DSD GD	NR5A1/ OTX2	c.991 − 1G > C (NR5A1)/p.P134R (OTX2)	Splice site/exon	Het	2020
		NR5A1/ PROP1	c.991 − 1G > C (NR5A1)/p.L102Cfs*8 (PROP1)	Splice site/exon	Het	
		SOS1	p.Y136H	Exon	Het	
Xu et al.^96^	Leydig cell hypoplasia	LHCGR	p.L153P	Exon	Hom	2020
Hassan et al.^97^	DASA	HSD17B3	p.Q148*	Exon	Hom	2020
		HSD17B3	p.Q148*			
		LHCGR	p.L104P			
Hughes et al.^98^	46, XY DSD (syndromic)	PPP1R12A	p.L924Rfs*14	Exon	Het	2020
			p.R504*			
			p.S692Ifs*2			
			p.R900*			
			p.E321Rfs*6			
			p.T397Hfs*42			
			p.K228*			
Altunoglu et al.^99^	46, XY DSD GD (syndromic)	PPP2R3C	p.S216_Y218dup	Exon	Hom	2021
			p.L193S			
			p.L103P			
Laochareonsuk et al.^100^	P450c17 deficiency	CYP17A1	p.Y329Fs/p.R358Q	Exon	Comp. Het	2021
Acar et al.^101^	PMDS	AMH	p.R439C	Exon	Hom	2021
			p.L115Tfs*58	Exon	Hom	
Akramov et al.^102^	Denys‐Drash syndrome (incomplete)	WT1	p.R369*	Exon	Het	2021
Edwards et al.^103^	Denys‐Drash syndrome	WT1	p.H445R	Exon	Het	2021
Özge et al.^104^	Mixed GD		Mosaic 45, X/47, XXY	Chromosome		2022
Wei et al.^105^	46, XY DSD GD	PPP2R3C	p.F229del/p.G417E	Exon	Comp. Het	2022
Cheng et al.^106^	46, XY DSD GD	MAP3K1	p.Q1007R	Exon	Het	2022
Yu et al.^107^	46, XY DSD GD	MAP3K1	p.K190N	Exon	Het	2022
Rjiba et al.^108^	46.XY DSD GD (syndromic)	NR0B1 dup	Xp 21.2 dup	CNV		2022
Baz‐Redón et al.^27^	46, XY DSD GD (Syndromic)	HHAT	p.M334K	Exon	Hom	2022
Nishi et al.^109^	46, XY DSD GD	NR0B1 dup	Xp 21.1 dup	CNV	N/A	2022
Sharif et al.^110^	Leydig cell hypoplasia	LHCGR	pL365Pfs*5	Exon	Hom	2022
Hong et al.^111^	PMDS	AMHR2	p.G40R/p.A408P	Exon	Comp. Het	2022
			p.G40R/p.A408P			
Nguyen et al.^112^	DASA	AR	p.C612S	Exon	Hem	2022
			p.L708V			
			p.F877C			
			p.L881M			
Aghaei et al.^113^	DASA	AR	p.L763V	Exon	Hem	2022
Çiftci et al.^114^	DASA	HSD17B3	c.673 − 1G > C	Splice site	Hom	2022
Gonçalves et al.^115^	DASA	HSD17B3	p.A203V/p.E215D	Exon	Comp. Het	2022
Sreenivasan et al.^116^	DASA	AR	Exon 2 dup	CNV	Hem	2022
Çelik et al.^117^	46, XY DSD w/heart defect	GATA4	p.T113P	Exon	Het	2022
	46, XY DSD		p.P163S			
Shichiri et al.^118^	46, XY DSD w/heart defect	GATA4	p.P163S	Exon	Het	2022
Francese‐Santos et al.^30^	46, XY DSD GD	*GK*, *TASL*, partial *TAB3* duplication	Xp21.2 Dup (w/o *NR0B1*)	CNV		2022
Calonga‐Solís et al.^119^	46, XY DSD GD w/heart defect	*MYRF*	p.Q838*	Exon	Het	2022
Xie et al.^120^	46, XY DSD GD	SRY	p.L94R	Exon	Hem	2023
Zhang et al.^167^	46, XY DSD GD	NR5A1	p.G22C	Exon	Het	2023
Harris et al.^121^	46, XY DSD GD (Syndromic)	PPP1R12A	p.*1031Lext*71	Exon	Het	2023
Chisato et al.^122^	46, XY DSD GD	SRY	p.S91C	Exon	Hem	2023
de Oliveira et al.^123^	46, XY DSD GD	DHX37	p.R308Q	Exon	Het	2023
		DHX37	p.V999M	Exon	Het	
		DHX37/NR5A1	p.V999M (DHX37)/p.S4* (NR5A1)	Exon	Het	
		DHX37/NR5A1	p.L467V (DHX37)/p.M98Qfs*45 (NR5A1)	Exon	Het	
Meinel et al.^124^	46, XY DSD GD	Xp21.2 Dup (w/NROB1)	Two region duplications, Two small deletions	CNV		2023
		Xp21.2 Triplication (w/o NROB1)	Xp Triplication	CNV		
Noveski et al.^125^	DASA	AR	c.−547C > T	5′ UTR	Het	2023
Garcia et al.^126^	DASA	HSD17B3	c.277 + 4A > T/p.E215D	Splice site/Exon	Comp. Het	2023
	DASA	HSD17B3	Dup. chr3:3158485‐39924901, Dup. chr16:15129940‐16363239	CNV		
Rashmi et al.^127^	DASA	CYP17A1	p.F54del	Exon	Hom	2023
Bergougnoux et al.^128^	DASA	HSD17B3	c.277 + 4A > T	Splice site	Het	2023
	DASA	HSD17B3	c.278 − 1G > C	Splice site	Het	
	DASA	AR	p.Q799E	Exon	Hom	
	DASA	SRD5A2	p.L152Yfs*8	Exon	Hom	
	PMDS	NR5A1	p.C13S	Exon	Het	
	46, XY DSD GD	NR5A1	p.Q357*	Exon	Het	
Rahim Karim et al. ^129^	46, XY DSD	NR5A1	p.R69P	Exon	Het	2023
Yu et al.^130^	46, XY DSD w/Micropenis and UDT	NR5A1	c.102+1G > C	Splice site	Het	2023
Del Gobbo et al.^199^	46, XY DSD	NR5A1	Intronic 2752 bp intron 4 insertion	Intron	Het	2023
Correa Brito et al.^131^	46, XY DSD w/Heart defect	MYRF	p.Trp322*	Exon	Het	2023
Jian‐Wu et al.^132^	46, XY DSD GD	NR5A1	p.Q42E	Exon	Het	2023
Aversa et al.^133^	46, XY DSD micropenis and UDT	GATA4	p.S224C	Exon	Het	2023

*Note*: Publications and case reports that reported genetic diagnoses in individuals with 46, XY DSD. The associated 46, XY DSD categories of the individual(s) are listed. Genes implicated in 46, XY DSD, variants reported, location of variants, mode of inheritance, and year of publications are shown.

Abbreviations: CNV, copy number variant; Comp., compound; DASA, androgen synthesis/action disorder; Del, deletion; Dup, duplication; GD, gonadal dysgenesis; Hem, hemizygote; Het, heterozygote; Hom, homozygote; OT, ovotesticular; PMDS, persistent mullerian duct syndrome; Trp, triplication; UDT, undescended testis; UTR, untranslated region.

**TABLE 2:** Cohort studies of 46, XY differences/disorders of sex development (DSD) cases from 2018 to 2023.

Publication	Cohort profile	46, XY DSD cohort size	% Diagnosed	Year
Buonocore et al.^134^	46, XY DSD	52	30.8	2019
Hughes et al.^135^	46, XY DSD and 46, XX DSD	73	34.2	2019
Xu et al.^77^	46, XY DSD and 46, XX DSD	96	46.9	2019
Yu et al.^136^	46, XY DSD	87	42.5	2021
Mazen et al.^137^	Chromosomal DSD, 46, XY DSD, and 46, XX DSD	100	33.7 Sanger	2021
			66.7 WES	
Ata et al.^138^	Chromosomal DSD, 46, XY DSD, and 46, XX DSD	143	31.4	2021
Miclea et al.^139^	Chromosomal DSD, 46, XY DSD, and 46, XX DSD	27	Gene panel (52.9)	2021
			aCGH (22)	
Leitao Braga et al.^140^	46, XY DSD syndromic and non‐syndromic small for gestational age children with hypospadias	41 NS	2.4	2021
		5 S	75	
Zidoune et al.^141^	46, XY DSD and 46, XX DSD	125	49.6	2022
Gomes et al.^142^	46, XY DSD (non‐syndromic)	263	59.3	2022
Globa et al.^143^	46, XY DSD	71	46.5	2022
Tang et al.^144^	46, XY DSD	178	35.96	2023

*Note*: Published cohort studies analyzing 46, XY DSD cases from 2018 to 2023. Cohort profile, year of publication, and size of 46, XY DSD cohort are shown with percentage of diagnosis from the 46, XY DSD cohort.

Abbreviations: NS, non‐syndromic; S, syndromic; WES, whole exome sequencing; aCGH, array comparative genomic hybridization.

### 4.1 The role of molecular and cytogenetics technologies in 46, XY DSD diagnosis

For individuals born with a DSD, confirming the karyotype/genetic sex ruling out chromosomal causes is an important step in the diagnostic process. Conventional karyotyping uses the visualization of chromosomes in metaphase^145^ and can detect aberrations to chromosome number and identify deletions, duplications, inversions, and translocations larger than 5 Mb. This technology continues to be of importance in DSD; however, it is unsuitable for smaller abnormalities, expensive, and slow because it relies on culturing cells. A more rapid cytogenetic technique is fluorescent in situ hybridization (FISH) that uses fluorescent probes complementary to specific chromosomal regions and can be used to identify the presence of *SRY* or to confirm aberrant sex chromosome or *SRY* translocation.^146^, ^147^ FISH does not rely on cell culture, although its application is limited as it requires a known target region. Quantitative fluorescence PCR (QF‐PCR) is another technology that uses short tandem repeats (STRs) to differentiate between chromosomes.^148^ Aside from the rapid turnaround time, QF‐PCR can detect almost 90% of clinically significant chromosomal abnormalities.^149^

Karyotyping, QF‐PCR, and FISH can establish genetic sex, aid in the classification of DSD, and detect aneuploidies and large structural rearrangements; however, detecting smaller copy number variations (CNVs) or InDels (insertions/deletions) requires technologies with higher resolution. Many sex‐determining genes, such as *SOX9*, *NROB1*, *WT1*, and *DMRT1*, are dose dependent with whole gene deletions or duplication causing a variety of 46, XY DSD phenotypes.^150^ Microarray‐based comparative genomic hybridization and mulitplex ligation dependent probe amplification (MLPA) technologies have been used to detect these variations. MLPA is a PCR‐based method that uses two oligonucleotide probes hybridized to a genomic region of interest.^151^, ^152^ CGH array uses oligonucleotide probes spread out through the whole genome or targeted to a specific genomic region.^153^, ^154^ It can detect sub‐microscopic copy number changes as small as 1 kb. It is slowly replacing conventional cytogenetics method,^155^ although limitations of this technology include an inability to detect balanced inversions or translocations, or to discern the exact location of duplicated regions. CGH array technologies have been widely used in DSD, and numerous reviews have discussed this in more detail.^156^, ^157^

### 4.2 Sequencing technologies in 46, XY DSD diagnosis

The detection of single‐nucleotide variants (SNVs) or InDels requires DNA sequencing technologies targeting either a single gene, a group or “panel” of genes, whole exome (WES), or even the whole genome (WGS). Sanger sequencing uses fluorescent chain–terminating dideoxy‐nucleotides and can provide a quick genetic diagnosis especially in cases where the phenotypic presentation is indicative of a single gene, such as the case for the *CYP21A2* gene in CAH cases.^6^ Sanger sequencing is still an important tool in the diagnosis process, including for confirmation of variants found in other technologies,^158^, ^159^, ^160^ and can be used in combination with enzyme digestion to target genes that have highly homologous pseudogenes that can interfere with alignment in MPS technologies.^161^

MPS, also known as next‐generation sequencing, enables the simultaneous sequencing of millions of DNA fragments. A variety of MPS technologies exist. Illumina MPS platforms use a method called sequencing by synthesis (SBS) where the incorporation of a nucleotide produces fluorescence that can be captured by small detection charged‐coupled device cameras.^162^ Here, DNA fragments annealed to adapters and immobilized in flow cells undergo amplification to form “clusters” of cloned DNA, the site of SBS.^162^ Other platforms, such as the Ion Torrent platform, use semiconductor chips to detect changes in pH/the release of hydrogen ions that occur when nucleotides are incorporated into a growing DNA strand during polymerization.^163^

**FIGURE 3:**
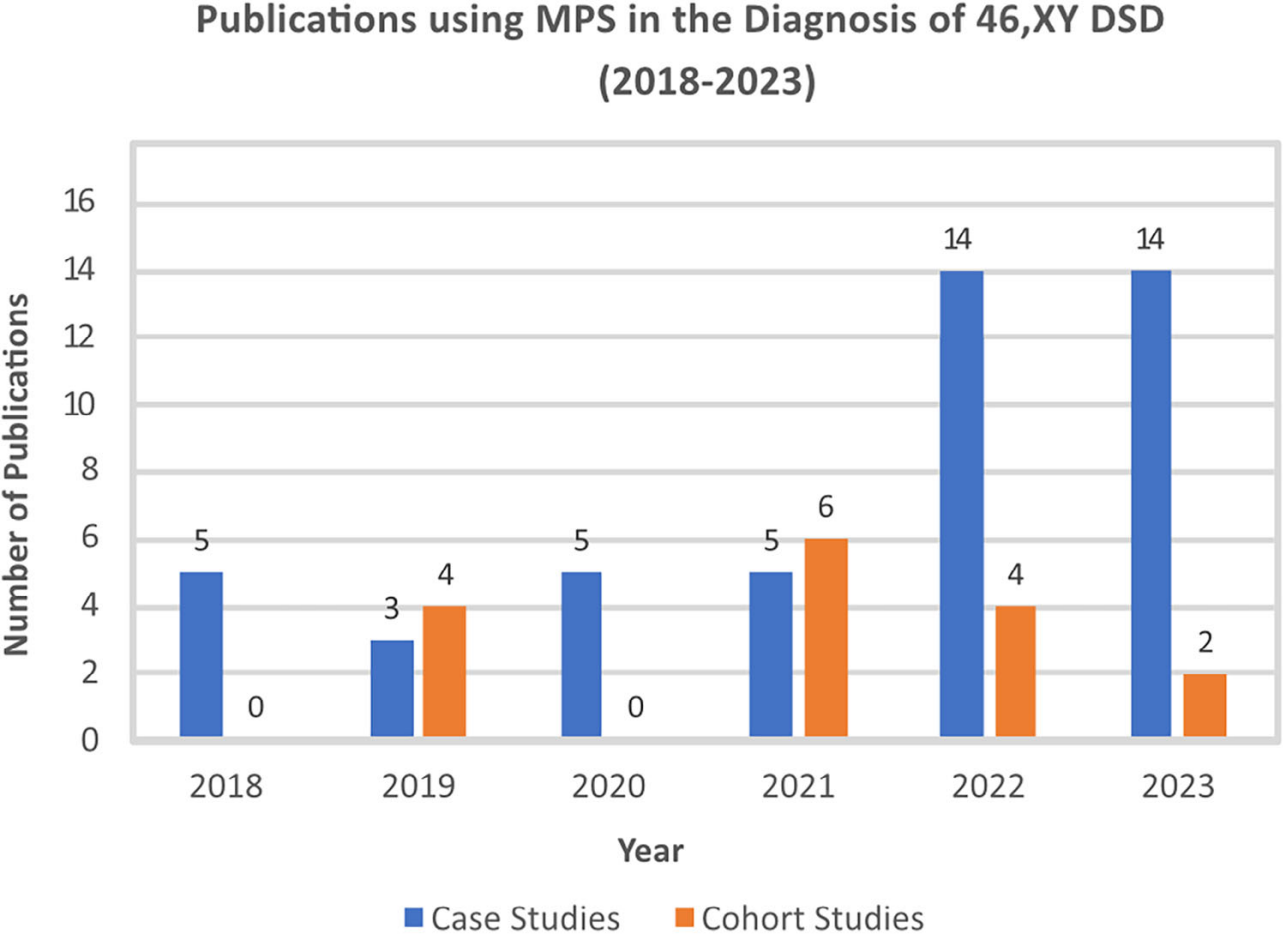
The number of publications using massively parallel sequencing (MPS) in the diagnosis of differences/disorders of sex development (DSD) (2018–2023). The use of MPS to find genetic variants in individuals with 46, XY DSD is increasing, especially in individual case reports.

**FIGURE 4:**
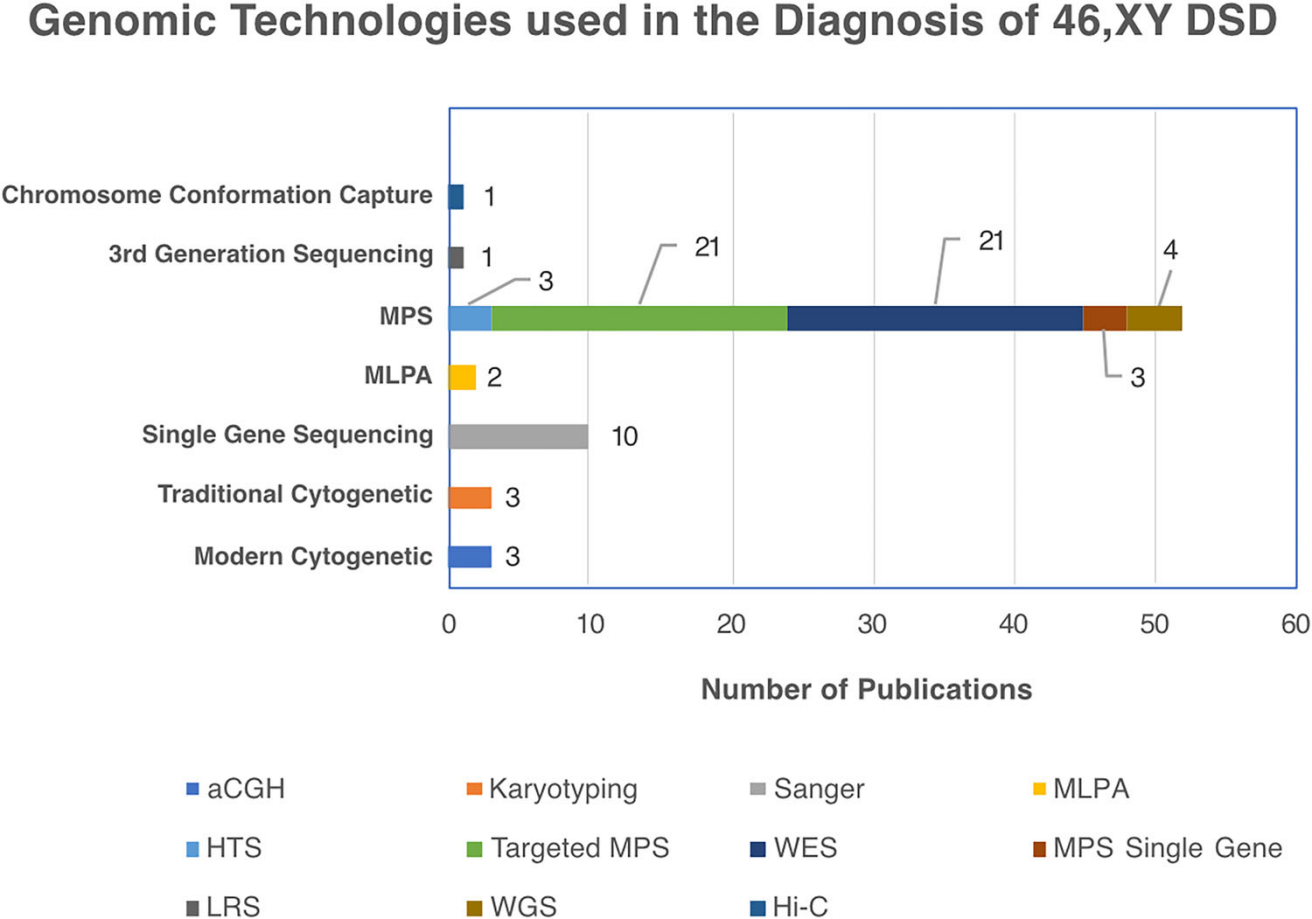
Genomic/genetic technologies used in published 46, XY differences/disorders of sex development (DSD studies from 2018 to 2023. aCGH, array‐based comparative genomic hybridization; LRS, long range sequencing; MLPA, multiplex ligation dependent probe amplification; MPS, massively parallel sequencing; WES, whole exome sequencing; WGS, whole genome sequencing.

MPS use in DSD diagnostics is increasing. In the last 5 years, we found the number of case reports using MPS increased from 5 studies in 2018 to 14 in 2023 (Figure 3), with cohort studies going from 4 publications in 2019 to 6 studies in 2021 (Figure 3). The Illumina MPS was the platform of choice in 91% of these studies. Ten years on from the first study that applied a targeted MPS gene panel to a DSD cohort,^164^ we found this technology is still relevant for DSD, with 21 studies in the last 5 years (Figure 4), ranging from 30 to 4800 genes, including up to 168 known DSD causative genes.

One of the disadvantages of targeting sequencing using gene panel is that if no causative variant is found, the data cannot be used for novel gene discovery. This can be overcome by using WES, where sequencing is targeted to all gene coding regions (exons), which account for about 2% of the whole genome. Indeed, in WES, the use of virtual panels to focus on genes relevant to a particular condition can enhance the analysis efficiency, while still providing sequencing data to widen the search in cases where no diagnosis is found. In 2018, just 2 case reports used WES to find a genetic diagnosis in DSD, whereas in 2022, as many as 10 publications reported the use of WES (Table 1). WES produces a wealth of genetic data, often revealing a multitude of variants that are likely to be benign or nondisease causing increasing the challenge of variants interpretation as discussed in a later section. Indeed, the manageable data size, reduced bioinformatics workload, and cost effectiveness as well as the depth of sequencing offered by targeted MPS gene panel approach^165^, ^166^ may explain its continued popularity compared with WES; as it still accounted for 50% of MPS in case studies and cohorts 2023.

MPS has enabled a rapid identification of numerous new variants in known DSD causing genes such as *DHH*,^81^ *NR5A1*,^167^ *HHAT*,^27^ *CYP17A1*,^100^ *AMRH2*,^91^ and *GATA4*.^117^ It has also revealed novel genes such as *SART3*,^168^ *DHX37*,^169^ and *ZNRF3* ^170^ and has led to genotype–phenotype expansion for several other genes. One example is the *PPP2R3C* gene. Being originally associated with syndromic 46, XY DSD by Guran et al.^89^ in individuals presenting with testicular dysgenesis and facial dysmorphism, myopathy, and skeletal abnormalities, variants in this gene have now been associated with a large degree of external genital phenotypes from complete female to ambiguous genitalia with or without ocular or muscular syndrome.^99^, ^171^, ^172^ WES has also revealed potential examples of oligogenicity in DSD (the contribution of multiple genes to a phenotype) such as digenic variants in *NR5A1* and *DHX37* ^41^ and multiple DSD genes with *NR5A1*.^173^ In other studies, potential modifiers have been proposed such as *OTX2* and *PROP1* variants, which may account for incomplete penetrance associated with an *NR5A1* variants.^78^

MPS of DSD cohorts has delivered genetic diagnostic rates between 2.4% and 66.7% (Table 2). This vast range may be explained by differing technologies, variant curation or by cohort inclusion criteria,^174^ where the inclusion of DSDs with a nongenetic causes, such as hypospadias, may reduce diagnostic findings. Increasing the size of the gene panel does not correlate with increased diagnostic rate (Table 2). WES is thought to deliver a diagnostic yield of 30%–45%^4^, ^5^ with recent DSD cohort studies reporting rates from 35.96% to 66.7% (Table 2).

Although targeted gene MPS and WES have led to significant increases in genetic diagnostic in DSD, few studies have provided a diagnosis rate greater than 50%, indicating that in many cases novel genetic causes remain to be found. Indeed, recent studies have indicated that the answers to these cases may lie in variants in noncoding regions of the genome or structural variations. Noncoding variants associated with DSD include single‐nucleotide changes, duplications, deletions, inversion, and translocations.^175^ Splice site variants in *NR5A1*,^78^, ^130^ *HSD17B*,^176^ and *AMRH2* ^85^ have also been reported, and recently, a diagnostic deep intronic change within the *AR* gene was found in two 46, XY sisters with AIS.^177^ Noncoding regions can also contain important *cis*‐regulatory regions such as enhancers. Several enhancers for *SOX9*, which reside far from the gene itself, have been shown to play an important role in the pathogenesis of 46, XY DSD (reviewed in Ohnesorg et al.^178^). WGS offers numerous advantages over WES such as identifying these noncoding genomic variants and eliminating the need for target enrichment, thereby reducing potential biases with a better detection of structural variants and complex genomic rearrangements.^179^ Despite these advantages, WGS is not yet widely applied to DSD. We found four studies that have employed WGS since 2018 in a total of six patients (Figure 4). One of these studies identified a pathogenic variant in the novel DSD gene *MYRF* ^119^ (Myelin regulatory factor). This gene encodes a transcription factor thought to be expressed in the early development of the genitourinary tract, which may regulate the *CITED2* gene.^119^ Another WGS study^98^ identified a variant in the newly recognized DSD gene *PPP1R12A* (protein phosphatase 1, regulatory subunit 12a). Variants in this gene cause both brain and urogenital malformations.^180^ The *PPP1R12A* gene encodes a targeting subunit of a myosin phosphatase complex.^181^ The role of the *PPP1R12A* gene in gonad development is currently unknown; however, a recent study^182^ using rat and mouse models found that PPP1R12A increases in phosphorylation by 4.7‐fold in response to LH stimulation. LH induces testosterone production in Leydig cells,^183^ providing some insight into a possible mechanism of PPP1R12A action. Both the *PPP1R12A* and *MYRF* variants identified in these studies are located in the coding regions and would have been identified using other MPS technologies such as WES. However, the other two WGS studies highlighted the utility of WGS in that they detected structural rearrangements in Xp21.2, affecting the *NR0B1* gene or surrounding locus,^30^, ^184^ aberrations unlikely to have been detected using standard WES analysis. WGS requires significant investment in storage as data generated can reach 120 GB per genome compared with 6 GB in WES or 1 GB in targeted sequencing.^185^ In addition, although WES will typically reveal about 50,000 variants (SNVs, large structural variants, and InDels less than 50 bp) in one individual, WGS can reveal as many as 3 million variants.^186^ This means that although costs of the technology itself may drop, the costs of a diagnostic WGS from an accredited lab are still often significantly higher than for WES due to the need to process, interpret, and curate more variants. Confidently and consistently interpreting variants in the noncoding regions of the genome can be challenging. To overcome this, bioinformatics tools (MutationTaster, CADD, and Genomiser) as well as curated databases (ClinVar, ENCODE, BluePrint, and FANTOM) have been developed to interpret noncoding variants.^187^

### 4.3 Understanding structural variations in 46, XY DSD

A key aspect of sex determination is the antagonistic interplay between testis and ovarian signaling pathways controlled by a precise regulation of gene expression, whereby even small changes to the dosage of key genes can cause imbalanced signaling and result in DSD.^16^ Changes to gene dosage can be caused by structural variations or CNVs that cover whole genes, regulatory regions, or change the genomic landscape. Although typically identified using Karyotyping, CGH array, or MLPA, structural variations can also be identified using WES and WGS. A 2022 study used WES data and a custom analysis pipeline that analyses read depth to identify a 43.6 kb duplication affecting the *AR* gene.^116^ A similar analysis was used to identify duplications of the chromosomal region 16q23.1q24.3, including the *WWOX* gene in two 46, XY DSD cases with micropenis and hypospadias.^188^ WGS was recently used to identify two large duplications and two small deletions in one individual and a triplication in another in the *NR0B1* containing the Xp21.2 genomic region associated with 46, XY GD.^184^

Structural variants can cause changes to 3D chromatin conformation. Topologically associated domains (TAD) are 3D organizational boundaries that create distinct regulatory segments in the genome and disruption to these can interfere with gene expression.^189^ Several technologies now exist to understand the interactions within the genome, such as Hi‐C technology, which uses a biotin linker to ligate and pull‐down crosslinked fragments followed by sequenced.^190^ The utility in diagnostics has been demonstrated in patients with developmental disorders where it has been used to resolve the precise locations of duplications, translocations, as well identifying breakpoints in a complex chromosomal rearrangement.^191^ In recent years, this technology has been applied to DSD for the first time. *SOX9* enhancers for the upstream of the gene (2Mb) underlie sex reversal when duplicated or deleted in humans and in mice.^178^, ^192^, ^193^ In silico Hi‐C analysis of this genomic region (17q24.3) has revealed that intra‐domain interactions in this region are critical for *SOX9* dynamic and cell‐specific expression, perhaps explaining part of the importance of these regions in DSD.^194^ In another study, combined WGS and Hi‐C analysis of the Xp21.2 *NROB1* locus in two unrelated patients with 46, XY GD found that disruptions created a novel chromatin domain (neo‐TAD) resulting in the hijacking of enhancers that leads to the upregulation of *NROB1*.^184^ Several limitations of Hi‐C currently reduce its widespread utility in DSD. It requires a large amount of reads in order to analyze chromatin interactions and is best employed on cells/tissue that are abundant and where the gene of interest is expressed.^195^ This is a major challenge in DSD where access to patient gonadal tissue is often limited.

## 5 CHALLENGES IN 46, XY DSD GENETIC DIAGNOSIS

### 5.1 Understanding variant pathogenicity

A challenge in any sequencing technology is interpreting the potential consequence of a given genomic variant. Variant curation and classification refer to the application of evidence‐based clinical interpretation and international standards for this exist, such as the American College of Medical Genetics (ACMG) guidelines,^196^ which have been employed in multiple publications included in this review.^142^, ^143^, ^144^, ^197^ These include the use of variant population frequency and genetic heterogeneity. Current population genomic datasets are often predominantly based on European ancestry, meaning that genomic analysis in underrepresented populations can yield lower diagnostic rates.^198^ Therefore, new approaches to variant curation and assigning pathogenicity are essential. Integrative “omics” approaches, such as combined genomics, transcriptomics, proteomics, metabolomics, and epigenomics, can be helpful in interpreting variant pathogenicity. RNA‐sequencing can reveal cryptic changes to gene expression or splicing—something that previously could only be inferred from prediction tools. This technology recently revealed that an *NR5A1* intronic structural variant found in a large family with individuals presenting variable 46, XY DSD phenotypes, such as hypospadias, reduced fertility, and dysgenic testes, caused due to nonsense‐mediated decay.^199^

Given that protein levels are more proximal to the phenotype of an individual than that of mRNAs, there is also a growing interest in integrating proteomics and genetics data.^200^ Quantitative proteomics via mass spectrometry has been used to analyze the impact of a novel splicing variant in the *CLBP* gene found in an individual with premature ovarian insufficiency,^201^ and to show that an SNV in *SART3* in patients with syndromic 46, XY DSD causes a reduction in SART3 protein in an unknown mechanism, thus providing evidence of pathogenicity for this novel DSD gene.^168^ As with Hi‐C and other technologies, the utility of multi‐omics approaches is sometimes limited in DSD due to the inaccessibility of the primarily affected tissues and the fact that many genes will only be expressed in these. Nevertheless, this barrier may be overcome in the future, for example, with the use of CRISPR technologies to drive the expression of unexpressed genes in cell culture as a way of assessing the effects of genomic variants on expression or splicing.^202^

Proving the pathogenicity of variants can sometimes also require functional studies. Traditionally, animal models have been used, although significant disparity between fetal gonad development in humans compared with mice means application can be limited.^203^ Confounding this is a lack of human fetal gonadal cell lines. Some recent inroads into this have been achieved with publications of human fetal datasets, including single‐cell RNA‐seq (scRNA‐seq) and ATAC‐seq from the human fetal gonads.^204^, ^205^, ^206^

Indeed, this human scRNA‐seq dataset has proven beneficial in assessing the pathogenicity of variants in novel genetic causes of DSD such as the *MYRF* gene. Truncating, missense, and frameshift variants in the novel *MYRF* gene were recently reported in individuals with 46, XY GD^119^, ^207^ and scRNA‐seq data from human fetal gonads showed high *MYRF* expression in coelomic epithelium cells during early bipotential gonad stages, suggesting a role in early gonad development, in‐line with being causative for GD.^119^, ^207^ This type of data and recent research to derive gonadal cell lineages from human stem cells provide a promising solution for disease modeling.^208^

### 5.2 Detecting mosaicisms

Genetic mosaicism refers to the presence of two or more genotypically distinct cell lineages that originate from the same zygote within an individual. There is increasing evidence that mosaic variants contribute to a significant number of congenital conditions,^209^ and it has long been appreciated that mosaic chromosomal aneuploidies cause DSD,^7^ suggesting that single‐gene variant mosaicisms are also likely to underlie a portion of undiagnosed DSD cases. The difficulty here is detecting these mosaic variants, as the proportion of affected cells may differ between tissues, and analysis of gonadal tissue is rarely feasible. A potential solution is to carry out genomic analysis on multiple tissues. For example, use of ectoderm derived tissues, such as hair follicles and buccal mucosa, has been used to complement mesoderm derived blood cells in detecting low‐level 18p tetrasomy,^210^ and saliva samples (containing both mesodermal and ectodermal derived cells) have been used to detect mosaic pathogenic CNVs not detected in blood samples to improve diagnostics for syndromic intellectual disability.^211^ Increasing sequencing depth can also improve sensitivity in detecting mosaic SNVs and InDels with WGS data found to be superior in detecting mosaic SNVs and CNVs due to its higher and more consistent coverage across the genome.^212^

### 5.3 Variable expressivity and incomplete penetrance

Both incomplete penetrance (where some individuals with a variant do not display the associated trait) and variable expressivity (where a variant is associated with a range of phenotypic manifestations) have been described in 46, XY DSD. The underlying cause of these phenomena is unknown, although studies using 46, XY DSD mouse models (such as B6 XYpos) suggest that differences in spatiotemporal expression during gonadal development may contribute.^213^ Furthermore, the stochastic nature of gene regulation (i.e., fluctuating or small numbers of regulatory molecules per cell) during male sexual development likely plays a role too, especially for genes where dosage provides a switch in determining whether testicular or ovarian development takes place. Examples of this are 46, XY DSD caused by mutations in *SRY* ^214^ or in *SOX9* regulatory regions, in which numerous cases of an affected child inheriting a variant from an unaffected parent of the same sex have been reported.^215^, ^216^, ^217^ Oligogenicity may also play a role, as has been postulated in individuals with variants in *NR5A1*,^218^ *HSD17B3*,^219^ *HSD3B2*,^220^ *MAMLD1*,^67^ and *GATA4*.^117^ MPS has played a role in understanding oligogenicity and uncovering additional variants in genes where the first candidate gene seemed insufficient to explain a 46, XY DSD phenotype. This is the case for some variants found in *MAMLD1* that could be found in normal population and have normal transcriptional activity.^69^ Therefore, a second variant may need to be identified that could contribute to the phenotype. These reported second hits and evidence of oligogenic inheritance that could cause DSD have been reviewed extensively elsewhere.^74^, ^221^ These phenomena cause challenges in diagnostics where segregation of a variant in a family may be important evidence for determining pathogenicity; thus, further work is required to understand the contribution of these processes to each DSD and to highlight any additional genetic or environmental factors that may contribute.

## 6 PROMISING FUTURE TECHNOLOGIES

### 6.1 Long read sequencing

Short read sequencing (SRS) technologies lack the power to accurately resolve complex or repetitive genomic regions or to easily detect large structural variants.^186^ Newer long read sequencing technologies (LRS), such as Oxford Nanopore Technologies’ (ONT) nanopore sequencing and Pacific Biosciences’ (PacBio) single‐molecule real‐time (SMRT) sequencing,^222^ hold huge promise in diagnostics. PacBio's LRS technology uses a single‐polymerase enzyme that emits light when a nucleotide is incorporated in the synthesis of complementary DNA strand,^223^ whereas Oxford nanopore measures the ionic current from a nucleotide that is present in a pore at a given moment.^224^ Compared with the 150 bp achieved in SRS, this third‐generation sequencing technology can produce reads up to tens of thousands bp long^225^ with the power and utility of this recently demonstrated in Telomere‐to‐Telomere sequencing of the human genome.^226^ Currently, LRS limitations include high error rates (between 1% and 5% per base for SMRT), compared with 0.3% in SRS^227^, although new PacBio HiFi technology has decreased the error rate to 0.1%–0.5%.^228^ Cost is also a consideration; where the investment cost is relatively low for ONTs LRS such as MinIon and GridIon, but the cost per genomic coverage is higher than for SRS technologies.^187^ In finding clinical variants, LRS technologies are ideal for detecting structural variants in highly repetitive regions of the genome, including in conditions where STR variations play a role.^229^ Although use in DSD diagnostics is not yet widespread, LRS was applied to DSD in 2023 when PacBio's HiFi platform was used to detect a heterozygous 2752 bp retroelement insertion in the *NR5A1* gene in a large four‐generation family with 46, XY DSD^199^ (Table 1). This technology promises to identify more diagnoses and in complex genomic regions such as the regulatory regions upstream of *SOX9*.

### 6.2 Artificial intelligence in 46, XY diagnosis

Much work is underway to develop sophisticated artificial Intelligence (AI), particularly deep learning algorithms, to process complex genomic data.^230^ These apply to various challenges such as variant calling, genome annotation, and variant classification.^230^ Notable tools, such as SpliceAI^231^ and CADD,^232^ have helped in determining genetic variants pathogenicity and recently, a deep learning model for analyzing proteome‐wide missense variants, AlphaMissense, has been developed to analyze proteome‐wide missense variants.^233^ Image analysis deep learning algorithms that combine facial image analysis AI with genomic data for accurate variant prioritization, such as PEDIA,^234^ could be applied to DSD where images and other medical records linked to genomic data could aid in rapid diagnosis. Although care must be taken to avoid bias introduced by reliance on existing data to train deep learning algorithms where various groups may be underrepresented,^230^ the potential of AI in diagnostics is clear. This potential has been recently demonstrated using the dynamic uncertain causality graph model that was found to have an accuracy of 94.1% in diagnosing patients with DSD, outperforming both interns (64.7%), and third‐year residents (77.1%).^235^

## 7 CONCLUSION

Testis and ovary development is underpinned by complex signaling that, when disrupted, can cause differences/disorders of sex developments. The genetic causes of these are highly varied, but the advances in massively parallel sequencing technologies have greatly facilitated genetic diagnosis of individuals with 46, XY differences/disorders of sex development, often a crucial step for optimal clinical care. The use of these technologies has increased significantly over the past 5 years as exemplified by published case reports and cohort studies. Recently, a new wave of genomic technologies, such as Hi‐C, long read sequencing technologies, and omics analyses, have begun to be applied to differences/disorders of sex development and hold promise to fill current diagnostic gaps. However, challenges still exist including access to affected tissues, detecting mosaicism, and interpreting variants especially when faced with variable expressivity and incomplete penetrance. Recent advances in artificial Intelligence deep learning algorithms and stem cell testis models may help to overcome these challenges and assist in closing the current diagnostic gaps for 46, XY differences/disorders of sex development.

## Data Availability

Data sharing is not applicable to this article as no new data were created or analyzed in this study.
